# Clonal and plasmid-mediated dissemination of CTX-M-14–producing *Escherichia coli* within a single cattle farm in Japan

**DOI:** 10.3389/fmicb.2026.1772995

**Published:** 2026-02-18

**Authors:** Ryuichi Nakano, Yuki Suzuki, Akiyo Nakano, Koichi Yamaguchi, Saori Horiuchi, Yasuo Ono, Hisakazu Yano

**Affiliations:** 1 Department of Microbiology and Infectious Diseases, Nara Medical University, Nara, Japan; 2 Faculty of Health and Medical Science, Teikyo Heisei University, Tokyo, Japan

**Keywords:** clonal transmission, CTX-M-14, ESBL-producing *Escherichia coli*, IncI1 plasmid, livestock–human interface, whole-genome sequencing

## Abstract

**Introduction:**

Extended-spectrum *β*-lactamase (ESBL)–producing *Escherichia coli* threatens humans and animals. *bla*_CTX-M-14_, which is prevalent in Asia, is mainly disseminated via incompatibility group I1 (IncI1) plasmids, as they can efficiently transfer across Enterobacterales. However, direct evidence linking livestock and farmers at the genomic level is limited, and our study addresses this concern.

**Methods:**

Fecal samples were collected in 2013 from one parent cattle, three calves, and a farmer on a cattle farm in southern Kyushu, Japan. ESBL-producing *E. coli* were identified and characterized using hybrid Illumina–Nanopore assemblies, resistance gene profiling, and plasmid replicon typing. Clonal relatedness was assessed using core-genome multilocus sequence typing and core single-nucleotide polymorphism (SNP) analysis. Conjugation assays were used to evaluate plasmid transferability.

**Results:**

Seven *bla*_CTX-M-14_-positive *E. coli* isolates representing five sequence types (STs) were recovered. Two ST533 isolates from parent cattle and calf 1 were identical by core-genome ST and differed by only one core SNP, indicating recent clonal transmission. Additionally, ST1148, ST1261, and ST1431 were isolated from other calves, while ST448 isolates from the farmer and calf 3 exhibited a large genetic distance (3,891 core SNPs) and distinct cgSTs. All *bla*_CTX-M-14_ genes were located on conserved IncI1 plasmids of approximately 114 kb, showing >99.9% sequence identity, including the IncI1 plasmid. Conjugation frequencies ranged from 10^−2^ to 10^−3^. Other resistance genes, including *tet(A)* and *mcr-3.1*, were encoded on separate plasmids.

**Conclusion:**

These findings underscore the possible role of IncI1 plasmids in bridging resistance gene flow across host boundaries and emphasize the benefit of integrated One Health genomic surveillance to monitor and mitigate antimicrobial resistance transmission.

## 1 Introduction

Extended-spectrum *β*-lactamase (ESBL)–producing *Escherichia coli* are a major global health concern, as they undermine the efficacy of third-generation cephalosporins in both human and veterinary medicine (Tacconelli et al., 2018; Castanheira et al., 2021). Among ESBLs, CTX-M enzymes have become predominant worldwide (Bonnet, 2004; Bush and Bradford, 2020), with CTX-M-14 being one of the most frequently reported variants. In Japan, *bla*_CTX-M-14_ has been repeatedly identified in both clinical and livestock isolates, indicating its wide establishment across reservoirs (Kosai et al., 2020; Masui et al., 2022; Nakano et al., 2023). Similar trends have been observed in other Asian countries, highlighting their regional significance (Liao et al., 2015; Bevan et al., 2017).

*bla*_CTX-M-14_ is frequently encoded on plasmids and can disseminate through horizontal gene transfer among Enterobacteriaceae (Cantón et al., 2012; Yu et al., 2024). Particularly, incompatibility group I1 (IncI1) plasmids serve as key vectors; they are characterized by high conjugation efficiency, broad host range, and remarkable stability, facilitating their persistence and dissemination (Carattoli, 2009; Wong et al., 2016; Villa and Carattoli, 2020; Yu et al., 2024). These features make IncI1 plasmids highly effective vehicles for interlineage and interhost transmission of resistance genes.

Whole-genome sequencing (WGS)-based approaches, such as core-genome multilocus sequence typing (cgMLST) and core single-nucleotide polymorphism (core-SNP) analysis, allow discrimination of recent clonal transmission from coincidental sequence type (ST) overlap (Yan et al., 2023; Kavanagh et al., 2024). Studies applying these tools often demonstrate that human and animal isolates from the same farm cluster genetically or share nearly identical plasmids, supporting the potential for interspecies exchange (de Been et al., 2014; Aldea et al., 2022). However, definitive one-to-one evidence of direct transmission, such as ≤ 0–5 SNP differences between a farmer and their livestock, remains rare.

Previously, we reported the genetic relatedness of third-generation cephalosporin-resistant *E. coli* isolated from livestock, farmers, and patients in southern Kyushu, Japan (Nakano et al., 2023). The study demonstrated the occurrence of resistant *E. coli* across multiple hosts and highlighted the potential for transmission between human and animal populations. However, the relative contribution of direct clonal spread vs. plasmid-mediated transfer remained unclear.

In this study, using high-resolution whole-genome sequencing approaches, we aimed to investigate the clonal relatedness and plasmid characteristics of *bla*_CTX-M-14_-positive *E. coli* isolated from cattle and a farm worker on a single farm in Japan. Using a combination of antimicrobial susceptibility testing, hybrid WGS, cgMLST, core-SNP analysis, and plasmid characterization, we (i) determined the clonal relatedness between isolates from cattle and farmer, (ii) characterized the genomic features of *bla*_CTX-M-14_–encoding plasmids, and (iii) evaluated the conjugative transferability of these plasmids. By providing genome-level evidence of both clonal dissemination among livestock and the sharing of nearly identical IncI1 plasmids between livestock and a human host, this study extends previous findings and contributes to a deeper understanding of ESBL transmission dynamics in the farm environment.

## 2 Materials and methods

### 2.1 Isolation of third-generation cephalosporin-resistant *E. coli* from cattle and farmer

Fecal samples were collected from cattle and a farmer on a single cattle farm in the southern part of Kyushu Island, a major cattle farming area in Japan, in 2013. The fecal samples were directly inoculated onto deoxycholate-hydrogen sulfide-lactose agar plates and incubated at 37 °C for 24 h. Three colonies were randomly selected, and the species were identified using matrix-assisted laser desorption ionization-time-of-flight mass spectrometry (Vitek MS system; bioMérieux, Co., Ltd.). The isolates identified as *E. coli* were used for analyses. The Ethical Review Committee of the Teikyo University School of Medicine (No. 13–118) approved the study protocol, and the farmer provided written informed consent to participate in the study.

### 2.2 Antimicrobial susceptibility testing and detection of ESBL genes

Antimicrobial susceptibility of the isolates was determined through agar microdilution according to the Clinical & Laboratory Standards Institute guidelines, and quality control was performed using the reference strain *E. coli* ATCC 25922 (CLSI, 2025). The presence of ESBL genes in the third-generation cephalosporin-resistant *E. coli* isolates was identified using multiplex PCR (Dallenne et al., 2010). Gene-specific PCR was performed to identify the genotype, and the amplified products were confirmed using DNA sequencing (Ohnishi et al., 2013). Sequence alignment and analysis were performed on the National Center for Biotechnology Information website using the Basic Local Alignment Search tool (Altschul et al., 1990)^1^.

### 2.3 WGS and genomic analysis

Genomic DNA was extracted using the QIAGEN Genomic-tip 500/G kit (Qiagen, Germany). WGS was performed on both the Illumina MiSeq platform (Illumina Inc., USA) and Oxford Nanopore MinION platform (Oxford Nanopore Technologies, UK). Hybrid *de novo* assemblies were generated using Unicycler v0.5.0 (Wick et al., 2017), and genome annotation was conducted via DFAST v1.6.0 on default parameters.

Plasmid replicon types were determined with PlasmidFinder, and plasmid/genome structures were compared and visualized using Easyfig v2.2.2. Antimicrobial resistance genes were identified using ResFinder^2^ with thresholds ≥90% identity and ≥60% minimum length. Multilocus sequence typing (MLST) of *E. coli* isolates was performed, and STs were assigned using the PubMLST database^3^ (Wirth et al., 2006). Pairwise nucleotide identity among *bla*_CTX-M-14_-encoding plasmids was evaluated using the JSpeciesWS online platform^4^ (Richter and Rosselló-Móra, 2009). Average Nucleotide Identity based on MUMmer (ANIm) was calculated for all plasmid pairs under default settings.

### 2.4 cgMLST

cgMLST was performed using cgMLSTFinder 1.2 (Center for Genomic Epidemiology).^5^ Assembled genomes of all isolates were submitted to the pipeline, and allele calling was performed against the *E. coli* cgMLST scheme (2,513 loci). The output data included the total number of called alleles, ST assignments [core-genome Sequence Type (cgST)], and allelic distances between isolates. A minimum-spanning tree was constructed from the cgMLST allelic profiles using GrapeTree v2.2 (Zhou et al., 2018). The resulting phylogenetic relationships were further visualized and annotated with FigTree v1.4.4.^6^ Isolates with identical cgSTs and zero allelic distance were considered clonally indistinguishable, whereas those with small allelic differences (≤10 alleles) were regarded as highly related, according to previously described criteria (Zhou et al., 2021).

### 2.5 Chromosomal core-SNP analysis

Chromosomal core-SNP analysis was performed to assess the genetic relatedness. Therefore, plasmid sequences were excluded, and only chromosomal sequences were used. Sequence reads were mapped to the *E. coli* TK3888 genome (GenBank accession no. AP044770), which was used as the reference, using the CLC Genomics Workbench v24.0 (Qiagen) with default mapping parameters. To ensure accuracy, SNPs were called under the following thresholds: minimum coverage of 10×, minimum variant frequency of 90%, and minimum base quality score of 20. Putative SNPs located in repetitive or low-complexity regions were excluded, and only high-confidence SNPs in conserved chromosomal regions were retained for downstream analyses.

An SNP alignment was generated from all isolates, and a maximum-likelihood phylogenetic tree was constructed in CLC Genomics Workbench using the Jukes–Cantor model with 1,000 bootstrap replicates. Pairwise SNP distances were also calculated to determine the degree of genetic relatedness between isolates. Based on prior studies on Enterobacteriaceae, isolates differing by 0–5 core-SNPs were considered clonally identical, whereas larger distances (e.g., >15–25 core-SNPs) were interpreted cautiously as indicative of more distant relationships (Dallman et al., 2015; Ludden et al., 2021).

### 2.6 Plasmid conjugation assay

Conjugation experiments were performed using the broth mating method with *E. coli* J53 (sodium azide–resistant) as the recipient, as previously described (Masui et al., 2022). Briefly, donor and recipient strains were grown to the exponential phase in Luria–Bertani (LB) broth, mixed at a 1:1 ratio (vol/vol), and incubated overnight at 37 °C without shaking. After which, aliquots of the mating mixtures were plated onto LB agar supplemented with cefpodoxime (8 μg/mL) and sodium azide (100 μg/mL) to select for transconjugants. The presence of the resistance gene *bla*_CTX-M-14_ in transconjugants was confirmed using PCR. Conjugation frequency was calculated as the number of transconjugant colonies (cfu/mL) divided by the total number of recipient cells (cfu/mL).

## 3 Results

### 3.1 Characteristics of ESBL-producing *E. coli* isolates from cattle and farmer

**Figure 1:**
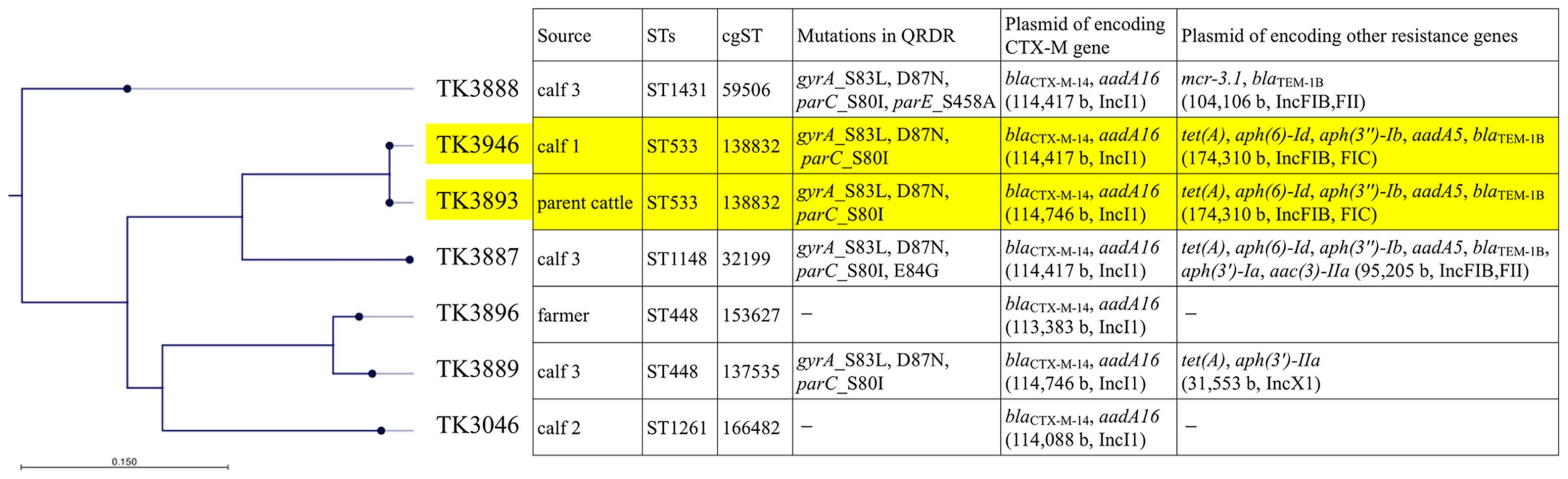
Core-genome SNP-based phylogenetic tree showing the isolation source, sequence types, mutations in QRDR, and plasmid contents of *E. coli* from parent cattle, calves, and a farmer. The tree was constructed based on core-genome SNPs using the General Time Reversible (GTR) model with 1,000 bootstrap replicates. The accompanying metadata matrix displays relevant genomic and phenotypic attributes for each isolate. The two *E. coli* isolates belonging to ST533 (from the parent cattle and calf 1), which showed clonal identity (one SNP difference and identical cgST), are highlighted with a yellow background to emphasize their close genetic relatedness. SNP, single-nucleotide polymorphism; QRDR, quinolone resistance–determining region; ST, sequence type; cgST, core genome ST.

Overall, 15 isolates (three each from one parent cattle, three calves, and the farmer) were obtained and identified as 13 *E. coli* and 2 *Klebsiella pneumoniae* isolates. Among them, seven *E. coli* isolates exhibited resistance to third-generation cephalosporins with an ESBL phenotype (Table 1). One isolate each was recovered from the parent cattle, two calves, and the farmer, whereas three isolates were obtained from the remaining calf. These seven isolates displayed diverse susceptibility profiles to other antimicrobials. Five isolates were resistant to levofloxacin and carried mutations in the quinolone resistance-determining regions of *gyrA* and *parC* (Figure 1). Four isolates carried *tet(A)* and exhibited resistance to tetracycline, two of which also carried *aph(3′)-Ia* or *aph(3′)-IIa* and were resistant to kanamycin. All seven isolates harbored the *bla*_CTX-M-14_ gene. Additionally, two isolates obtained from calf 3 carried further resistance determinants: TK3887 harbored *aac(3)-IIa* and was resistant to gentamicin, whereas TK3888 carried *mcr-3.1* and was resistant to colistin.

**Table 1:** Summary of genomic features, resistance determinants, and conjugation profiles of CTX-M-14–producing *Escherichia coli.*

Strains	Source	Sequence types	cgST	CTX-M gene	Other resistance genes	MIC (μg/mL)*^a^*										Transconjugant profile			
						CTX	CTX/CLA^b^	CAZ	CMZ	AZT	LEV	TET	KAN	GEN	COL	Transfer frequencies	Plasmid replicon type	Resistance genes	Plasmid size
TK3893	parent cattle	ST533	138,832	CTX-M-14	*tet(A), aph(6)-Id, aph(3″)-Ib, aadA5, bla*_TEM-1B_	16	0.125	2	1	2	8	>256	2	1	1	3.2 × 10^−3^	I1	CTX-M-14	114,746^b^
TK3946	calf 1	ST533	138,832	CTX-M-14	*tet(A), aph(6)-Id, aph(3″)-Ib, aadA5, bla*_TEM-1B_	8	≦0.06	2	1	2	16	>256	2	1	1	9.1 × 10^−3^	I1	CTX-M-14	114,417^b^
TK3046	calf 2	ST1261	166,482	CTX-M-14	-	8	≦0.06	2	0.5	2	≦0.06	8	2	0.5	1	3.1 × 10^−3^	I1	CTX-M-14	114,088^b^
TK3887	calf 3	ST1148	32,199	CTX-M-14	*tet(A), aph(6)-Id, aph(3″)-Ib, aadA5, bla*_TEM-1B_ *, aph(3′)-Ia, aac(3)-IIa*	16	0.125	2	1	4	64	>256	>256	128	1	3.3 × 10^−3^	I1	CTX-M-14	114,417^b^
TK3888	calf 3	ST1431	59,506	CTX-M-14	*mcr-3.1, bla*_TEM-1B_	16	≦0.06	2	0.5	2	16	16	2	1	16	1.6 × 10^−2^	I1	CTX-M-14	114,417^b^
TK3889	calf 3	ST448	137,535	CTX-M-14	*tet(A), aph(3′)-IIa*	8	≦0.06	1	0.5	1	16	>256	128	1	1	2.6 × 10^−3^	I1	CTX-M-14	114,746^b^
TK3896	farmer	ST448	153,627	CTX-M-14	-	16	≦0.06	2	0.5	2	≦0.06	8	2	0.5	1	7.2 × 10^−2^	I1	CTX-M-14	113,383^b^

^a^Antibiotics: CTX, cefotaxime; CLA, clavulanic acid; CAZ, ceftazidime; CMZ, cefmetazole; AZT, aztreonam; LEV, levofloxacin; TET, Tetracycline; KAN, Kanamycin; GEN, gentamicin; COL, colistin. ^b^MICs were determined in the presence of clavulanic acid (5 μg/mL). cgST, core-genome Sequence Type.

MLST analysis revealed five distinct STs among the seven isolates (Table 1). Notably, the three isolates recovered from calf 3 belonged to different STs. Shared STs were identified between the parent cattle and calf 1 (ST533), as well as between calf 3 and the farmer (ST448).

### 3.2 Clonal relatedness of *E. coli* isolates between cattle and farmer

To investigate the genetic relatedness of the isolates, both cgSNP analysis and cgMLST were performed (Table 1; Figure 1). The two ST533 isolates from the parent cattle (TK3893) and calf 1 (TK3946) differed by only one SNP in the core genome, strongly suggesting clonal identity. Consistently, both isolates were assigned to the same cgST (138832), further confirming their close genetic relationship. This represents a direct example of clonal dissemination between livestock individuals within the same farm.

Contrastingly, ST448 isolates from calf 3 (TK3889) and the farmer (TK3896) differed by 3,891 core SNPs and had distinct cgSTs (137,535 and 153,627), showing a distant relation, despite their sharing of the same ST (Supplementary Figure 1). Other isolates (ST1261, ST1148, and ST1431) differed by over 15,000 SNPs with distinct cgSTs, consistent with independent lineages. The three isolates from calf 3 belonged to different STs and cgSTs, indicating coexistence of multiple unrelated *bla*_CTX-M-14_-positive *E. coli* lineages within a single animal.

These findings highlight clonal dissemination among livestock (ST533) and demonstrate that shared STs alone (e.g., ST448) do not necessarily indicate recent transmission, underscoring the value of integrating SNP and cgMLST analyses.

### 3.3 Genomic features of CTX-M-14–encoding plasmids

**Figure 2:**
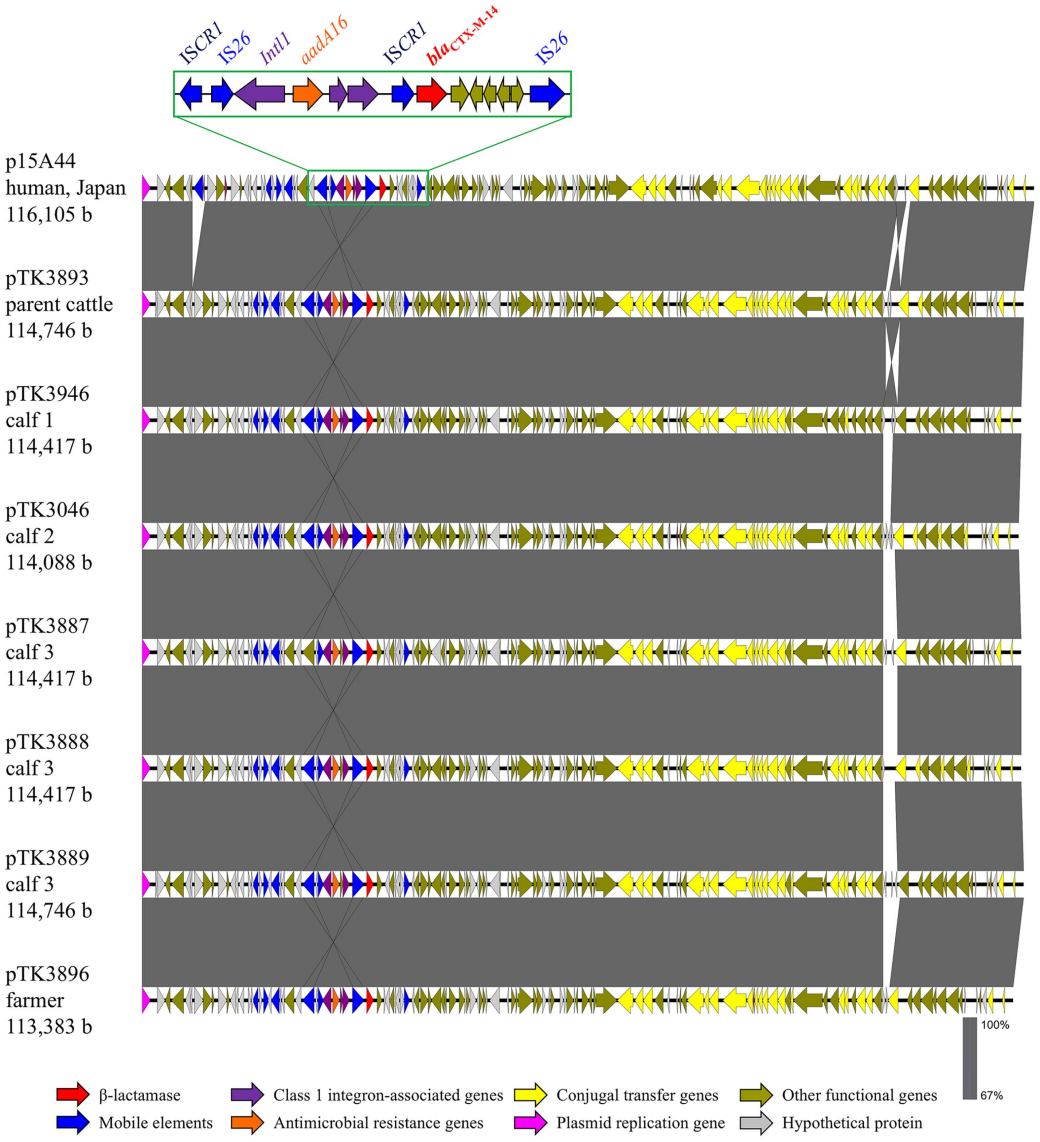
Linear comparison of *bla*_CTX-M-14_-carrying plasmid sequences from parent cattle, calves, and a farmer with plasmid p15A44 in Japan, and exposition of the detailed genetic structures around *bla*_CTX-M-14_. A legend explains the color code for gene functions. A magnified gene cluster with labels illustrates key resistance elements. Gray shading indicates sequence similarity, and sequence sizes are shown in base pairs. The GenBank accession numbers were: p15A44 (LC567051), pTK3893 (LC903451), pTK3946 (LC903453), pTK3046 (AP044764), pTK3887 (AP044768), pTK3888 (AP044771), pTK3889 (AP045037), and pTK3896 (AP045044).

Hybrid assembly and comparative analysis revealed that the *bla*_CTX-M-14_ gene in all positive isolates was consistently located on highly similar IncI1-type plasmids of approximately 114 kb in size (Figure 2). These plasmids exhibited a conserved backbone structure with only minor variations in accessory regions. Pairwise ANIm analysis using JSpeciesWS demonstrated extremely high sequence similarity among the seven IncI1 plasmids, with ANIm values ranging from 99.98 to 100%, and alignment coverage between 99.1 and 100% (Supplementary Table 1). These results clearly indicate that nearly identical *bla*_CTX-M-14_-encoding IncI1 plasmids were shared among the cattle and farmer within the same farm.

No additional resistance genes were encoded on these IncI1 plasmids, indicating that they primarily functioned as dedicated vectors for the dissemination of *bla*_CTX-M-14_. Other resistance determinants—*mcr-3.1*, *aac(3)-IIa*, *aph* variants, *tet(A)*, and *bla*_TEM-1B_—were carried on separate plasmids belonging to different incompatibility groups (e.g., IncFIB/FII, IncFIB/FIC, IncX1; Figure 1), highlighting the diversity of plasmid backgrounds contributing to multidrug resistance within the same farm.

Notably, the two clonally identical ST533 isolates from the parent cattle (TK3893) and calf 1 (TK3946) carried indistinguishable IncI1 plasmids, as well as nearly identical IncFIB/FIC plasmids encoding *tet(A)* and aminoglycoside resistance genes. Moreover, the *bla*_CTX-M-14_-encoding IncI1 plasmids exhibited high sequence similarity (>99.9%) to an IncI1 plasmid deposited in GenBank (accession no. LC567051), based on BLAST analysis (Figure 2). This reference plasmid was isolated from a clinical *E. coli* strain in Japan in 2015. This finding is consistent with the possibility that highly conserved plasmids can disseminate across human and livestock reservoirs.

### 3.4 Conjugative transferability of CTX-M-14 plasmids

Conjugation experiments were performed with all seven CTX-M-14–producing *E. coli* isolates. The transfer frequencies of the plasmids ranged from 10^−2^ to 10^−3^ per recipient, indicating a relatively high conjugation potential (Table 1). All transconjugants consistently carried only the *bla*_CTX-M-14_–encoding IncI1 plasmid, whereas plasmids harboring additional resistance determinants, such as *aac(3)-IIa* and *mcr-3.1*, were not co-transferred. The antimicrobial susceptibility profiles of the transconjugants were uniform, and their MIC values for third-generation cephalosporins were comparable across all donor strains (Supplementary Table 2).

## 4 Discussion

### 4.1 Presence of CTX-M-14–producing *E. coli* in cattle and farmer

To the best of our knowledge, this is the first report in Japan providing genome-level evidence that nearly identical IncI1 plasmids carrying *bla*_CTX-M-14_ were shared between humans and animals in a farm. CTX-M-14–producing *E. coli* are frequently isolated from livestock (Hayashi et al., 2018; Nakano et al., 2023) and prevalent among individuals (Yano et al., 2013; Komatsu et al., 2018; Masui et al., 2022). This suggests widespread dissemination across human and animal reservoirs. Similar patterns have been reported in other Asian countries, especially China, highlighting the regional significance of CTX-M-14 in both clinical and agricultural contexts (Liu et al., 2018; Zheng et al., 2019; Chen et al., 2024).

A major factor in this predominance is the association of *bla*_CTX-M-14_ with IncI1 plasmids, exhibiting high conjugation efficiency, broad host range within Enterobacterales, and stability during bacterial replication (Cottell et al., 2011; Di Pilato et al., 2019). These properties facilitate persistence and rapid dissemination across bacterial lineages, host species, and ecological niches (Beyrouthy et al., 2021). The strong linkage between *bla*_CTX-M-14_ and IncI1 plasmids likely underlies the successful establishment and widespread distribution of *bla*_CTX-M-14_-positive *E. coli* in both clinical and livestock environments.

Notably, among all isolates recovered from cattle and the farmer, *bla*_CTX-M-14_ was the only ESBL gene detected. The absence of other ESBL types within the same farm suggests a limited diversity of ESBL-producing *E. coli* at the time of sampling, which may reflect a relatively restricted introduction of ESBL determinants into this farm environment.

### 4.2 STs and clonal spread

cgSNP analysis revealed that *E. coli* isolates from the parent cattle and calf 1 belonging to ST533 differed by only a single SNP, indicating clonal identity. Consistent with this, cgMLST analysis noted that these two ST533 isolates shared the same cgST, further supporting their close genetic relationship. This represents an example in which a parent cattle and her calf on the same farm shared a clonally identical strain at the chromosomal level (0–1 core SNP difference), a finding that is biologically plausible given the close contact between animals. Such a precise one-to-one chromosomal match between a specific animal pair (parent cattle and calf) within the same farm has been infrequently reported; most previous studies have instead described low-SNP clusters involving multiple animals and/or humans within a shared farm or environmental setting (Pietsch et al., 2018; Peng et al., 2022; Bachmann et al., 2024).

The seven CTX-M-14–producing isolates encompassed five distinct STs (ST1431, ST533, ST1148, ST448, and ST1261), reflecting genetic diversity within the farm. The three isolates from calf 3 exhibited different STs, indicating multiple circulating lineages. Among these, ST533 has been frequently detected in livestock from both Asia and Europe, suggesting adaptation to animal hosts (Huber et al., 2013; Dahms et al., 2015). Conversely, ST448 has been detected in both humans and animals, highlighting its potential to cross host boundaries and disseminate between humans and animals (Blaak et al., 2014; Quiñones et al., 2020; Sivarajan et al., 2025).

**Figure 3:**
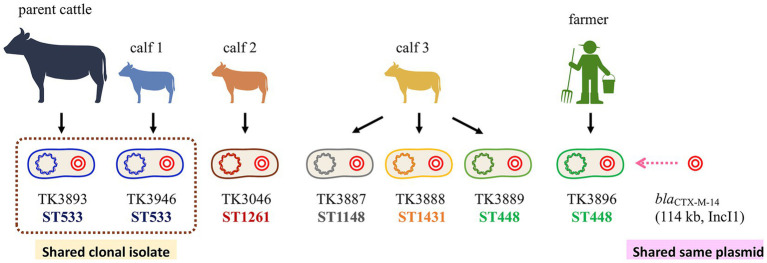
Summary of clonal and plasmid-mediated dissemination of *bla*_CTX-M-14–_positive *E. coli* on a cattle farm. Schematic summary of genomic relationships among *bla*_CTX-M-14_–positive *E. coli* isolates from cattle and a farmer in 2013. Core-genome SNP analysis identified a clonally identical ST533 pair from a parent cattle and its calf (0–1 SNP difference), consistent with recent clonal transmission. In contrast, distinct *E. coli* lineages harbored highly conserved IncI1 *bla*_CTX-M-14_–encoding plasmids (>99.9% sequence identity), suggesting dissemination of closely related plasmids across hosts and bacterial backgrounds. High conjugative transferability supports the potential role of these plasmids in ESBL spread at the livestock–human interface.

The identification of clonally identical ST533 isolates between the parent cattle and calf 1, supported by both SNP and cgMLST data, strongly indicates recent transmission and local clonal expansion within the livestock population. However, the ST448 isolates from calf 3 and the farmer differed by more than 3,800 core SNPs. These findings underscore the utility of high-resolution genomic analysis in detecting direct animal-to-human transmission, which may be difficult to infer from conventional typing methods. Thus, chromosomal SNP analysis demonstrated that the clonal spread of *bla*_CTX-M-14_-positive *E. coli* occurs independently of plasmid transfer, emphasizing the dual mechanisms whereby resistance persists and disseminates on farms. These relationships are schematically summarized in Figure 3.

### 4.3 Dissemination of identical CTX-M-14 plasmids

The transfer frequencies of IncI1 plasmids have been reported at levels of 10^−2^–10^−3^ per donor cell, which is considerably higher than those of many other plasmid types. Additionally, they stably persist without imposing a significant fitness cost on the host bacterium (Carattoli, 2009; Rozwandowicz et al., 2018). These properties facilitate the long-term maintenance and interspecies transfer of *bla*_CTX-M-14_.

Notably, we found that the 99.9% similarity between the plasmids detected in this study and those isolated in Japan further underscores the close genetic relationship between plasmids circulating in human and livestock populations. Therefore, IncI1 plasmids act as a “mobile resistome,” linking bacterial communities across different reservoirs. Similar observations have been reported in other countries, where IncI1 plasmids carrying *bla*_CTX-M-14_ were found to disseminate across diverse Enterobacterales lineages and between human and animal hosts (Liao et al., 2015; Yu et al., 2024). Our results reinforce the fact that plasmid-mediated dissemination is a major driver of ESBL spread in both clinical and agricultural settings.

### 4.4 Additional resistance determinants of concern

In addition, some isolates carried other clinically important resistance genes. One calf-derived isolate harbored *aac(3)-IIa* (gentamicin resistance), and another carried *mcr-3.1* (colistin resistance). The presence of *mcr* genes in livestock is concerning, as colistin is a last-resort antimicrobial for multidrug-resistant Gram-negative infections (Liu et al., 2016; Yin et al., 2017; Wang et al., 2018). *Tet(A)* and *aph(3′)* genes, conferring resistance to tetracyclines and kanamycin, respectively, were also detected, reflecting selective pressure from long-standing antimicrobial use in livestock (Van Boeckel et al., 2019). The coexistence of *bla*_CTX-M-14_ with additional resistance determinants on separate mobile elements may facilitate co-selection and persistence of multidrug resistance. These findings underscore the public health risk of accumulating multiple resistance genes in livestock-associated *E. coli* and highlight the need for ongoing genomic surveillance within a One Health framework.

### 4.5 Limitations and implications

This study has some limitations. First, the sampling was restricted to a single farm with a limited number of cattle and a single participant, which may not represent broader epidemiological trends. Second, although clonal and plasmid sharing were observed, the precise transmission direction (human-to-animal, animal-to-human, or environment-mediated) could not be determined. Third, longitudinal and environmental sampling was not performed, preventing assessment of temporal dynamics and external sources. Fourth, detailed epidemiological metadata such as the animal age, origin, housing conditions, or duration of cohabitation are lacking from data collected in this study, limiting our ability to fully reconstruct transmission pathways or exclude environmental or external sources. Finally, we did not investigate the detailed structural plasticity of the plasmids, such as transposable elements or recombination events.

## 5 Conclusion

The identification of nearly identical plasmids in both cattle and human hosts suggests that plasmid-mediated ESBL resistance traverses host boundaries in agricultural settings, reinforcing the importance of a One Health perspective in surveillance. Additionally, the similarity to plasmids found in clinical isolates indicates that livestock may act as microbial reservoirs, contributing to the wider dissemination of resistance determinants in human populations. Finally, the high transferability of IncI1 plasmids encoding *bla*_CTX-M-14_ further underlines the potential for rapid spread under antibiotic selection pressure.

## Data Availability

The datasets presented in this study can be found in online repositories. The names of the repository/repositories and accession number(s) can be found in the article/Supplementary material.

## Footnotes

1
https://blast.ncbi.nlm.nih.gov/Blast.cgi

2
https://genepi.food.dtu.dk/resfinder

3
https://pubmlst.org/organisms/escherichia-spp

4
https://jspecies.ribohost.com/jspeciesws

5
https://cge.food.dtu.dk/services/cgMLSTFinder/

6
http://tree.bio.ed.ac.uk/software/figtree/
