# Supplementary material for: Clonal and plasmid-mediated dissemination of CTX-M-14–producing Escherichia coli within a single cattle farm in Japan
Source: Front Microbiol. 2026 Feb 18;17:1772995. doi: 10.3389/fmicb.2026.1772995 (PMC12957190; doi:10.3389/fmicb.2026.1772995)
Supplement: Supplementary file 1 [file Data_Sheet_1.pdf]

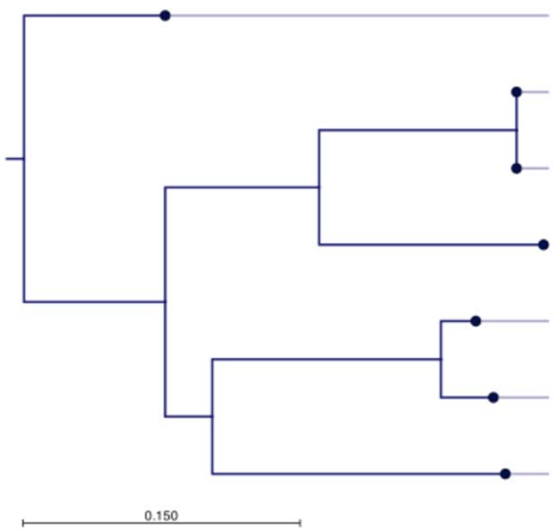

	TK3888	TK3946	TK3893	TK3887	TK3896	TK3889	TK3046
TK3888		20734	20735	21371	19311	19783	19603
TK3946	20734		1	15446	21116	21620	21422
TK3893	20735	1		15447	21117	21621	21423
TK3887	21371	15446	15447		21530	22184	22626
TK3896	19311	21116	21117	21530		3891	19155
TK3889	19783	21620	21621	22184	3891		19489
TK3046	19603	21422	21423	22626	19155	19489	

Supplementary Figure 1. Core-genome SNP phylogeny of CTX-M-14–producing *Escherichia coli* isolates and pairwise core-SNP distance heatmap. SNP, single-nucleotide polymorphism.

Supplementary Table 1. Pairwise average nucleotide identity (ANIm) and aligned percentage among $bla_{CTX\text{-}M\text{-}14}$-encoding IncI1 plasmids from from parent cattle, calves, and farmer

Plasmids [a] (GenBank acc. no.)	pTK3888	pTK3946	pTK3893	pTK3887	pTK3896	pTK3889	pTK3046
pTK3888 (AP044771)	*	99.99 [100.00]	99.99 [100.00]	99.98 [100.00]	99.99 [99.13]	99.99 [99.99]	99.98 [99.73]
pTK3946 (LC903453)	99.99 [100.00]	*	99.99 [100.00]	99.99 [99.99]	99.99 [99.12]	99.98 [100.00]	99.99 [99.74]
pTK3893 (LC903451)	99.99 [100.00]	99.99 [100.00]	*	99.99 [100.00]	100.00 [99.12]	99.99 [100.00]	99.98 [99.47]
pTK3887 (AP044768)	99.98 [100.00]	99.99 [99.99]	99.99 [100.00]	*	99.99 [99.11]	99.99 [100.00]	99.98 [99.73]
pTK3896 (AP045044)	99.99 [100.00]	99.99 [100.00]	100.00 [100.00]	99.99 [100.00]	*	99.99 [100.00]	99.99 [99.73]
pTK3889 (AP045037)	99.99 [99.99]	99.98 [99.99]	99.99 [99.99]	99.99 [100.00]	99.99 [99.12]	*	99.98 [99.46]
pTK3046 (AP044764)	99.98 [100.00]	99.99 [100.00]	99.98 [100.00]	99.98 [100.00]	99.99 [99.12]	99.98 [100.00]	*

[a] Values indicate pairwise ANIm (%) calculated using JSpecies (MUMmer-based). Numbers in brackets represent the proportion of the aligned region between each plasmid pair. All plasmids showed extremely high nucleotide identity (>99.9%) and near-complete alignment coverage (>99%), indicating their near-identical genomic structures.

Supplementary Table 2. Resistance genes and antimicrobial susceptibilities in transconjugants of CTX-M-14–producing *Escherichia coli*

Strains	Source	CTX-M gene	Other resistance genes	MIC (µg/ml) [a]									
				CTX	CTX /CLA[b]	CAZ	CMZ	AZT	LEV	TET	KAN	GEN	COL
pTK3893/J53	parent cattle	CTX-M-14	-	4	≦0.06	0.5	1	1	≦0.06	8	0.5	0.25	0.5
pTK3946/J53	calf 1	CTX-M-14	-	8	≦0.06	0.25	1	1	≦0.06	8	0.5	0.25	0.5
pTK3046/J53	calf 2	CTX-M-14	-	4	≦0.06	0.25	1	1	≦0.06	8	0.5	0.25	0.5
pTK3887/J53	calf 3	CTX-M-14	-	4	≦0.06	0.5	1	1	≦0.06	8	0.5	0.25	0.5
pTK3888/J53	calf 3	CTX-M-14	-	4	≦0.06	0.25	1	1	≦0.06	8	0.5	0.25	0.5
pTK3889/J53	calf 3	CTX-M-14	-	4	≦0.06	0.5	1	1	≦0.06	8	0.5	0.25	0.5
pTK3896/J53	farmer	CTX-M-14	-	4	≦0.06	0.5	1	1	≦0.06	8	0.5	0.25	0.5

[a] Antibiotics: CTX, cefotaxime; CLA, clavulanic acid; CAZ, ceftazidime; CMZ, cefmetazole; AZT, aztreonam; LEV, levofloxacin; TET, tetracycline; KAN, kanamycin; GEN, gentamicin; COL, colistin.

[b] MICs were determined in the presence of clavulanic acid (5 µg/mL).
